# The role of B-1 cells in cancer progression and anti-tumor immunity

**DOI:** 10.3389/fimmu.2024.1363176

**Published:** 2024-04-02

**Authors:** Nely Rodríguez-Zhurbenko, Ana M. Hernández

**Affiliations:** ^1^ Immunobiology Department, Immunology and Immunotherapy Division, Center of Molecular Immunology, Habana, Cuba; ^2^ Applied Genetics Group, Department of Biochemistry, Faculty of Biology, University of Habana, Habana, Cuba

**Keywords:** B-1 cells, natural antibodies, tumor immunity, immunosuppression, cancer

## Abstract

In recent years, in addition to the well-established role of T cells in controlling or promoting tumor growth, a new wave of research has demonstrated the active involvement of B cells in tumor immunity. B-cell subsets with distinct phenotypes and functions play various roles in tumor progression. Plasma cells and activated B cells have been linked to improved clinical outcomes in several types of cancer, whereas regulatory B cells have been associated with disease progression. However, we are only beginning to understand the role of a particular innate subset of B cells, referred to as B-1 cells, in cancer. Here, we summarize the characteristics of B-1 cells and review their ability to infiltrate tumors. We also describe the potential mechanisms through which B-1 cells suppress anti-tumor immune responses and promote tumor progression. Additionally, we highlight recent studies on the protective anti-tumor function of B-1 cells in both mouse models and humans. Understanding the functions of B-1 cells in tumor immunity could pave the way for designing more effective cancer immunotherapies.

## 1 Introduction

Tumors are complex cellular ecosystems consisting of malignant, immune, and stromal cells that dynamically interact with each other. The interactions between malignant and immune cells within the tumor microenvironment (TME) determine tumor growth or suppression. For decades, the study of tumor-infiltrating T lymphocytes (TIL-T) has been at the forefront of basic and translational research. Nevertheless, due to the considerable variability in the response rate to T-cell-based immunotherapy, additional immune cell populations have been recognized as modulators of tumor progression and as indicators of therapeutic response. An increasing body of research has focused on evaluating tumor-infiltrating B cells (TIL-B) as a potential tumor biomarker and a promising therapeutic target for combating tumors. Actually, TIL-Bs have shown significant predictive and prognostic value in the context of both traditional therapies and immune checkpoint blockade across various cancer types (1). B cells can play either a pro-tumor or anti-tumor role depending on their phenotype, function, and anatomic location within the tumor. B cells with immunosuppressive function, such as regulatory B cells (Bregs), are associated with reduced survival in cancer patients (2). Contrastingly, the presence of various types of effector B cells, such as germinal center-like follicular B cells, plasma cells, memory B cells, and antigen-presenting B cells within tertiary lymphoid structures (TLS), is associated with favorable outcomes in different human cancers (3–6). Among the various subsets of B cells infiltrating tumors, B-1 cells have emerged as a potential player in tumor immunity.

## 2 A brief overview of B-1 cells

B-1 cells represent a unique population that differs from conventional B-2 cells in developmental origin, phenotype, anatomic location, and function (7, 8). In mice, B-1 cells primarily reside in the body cavities and are present at a low frequency in the spleen and bone marrow. They are identified as CD19^high^B220^low^CD23-CD43+IgM^high^IgD^low^ (9), although the expression of other molecules further divides the B-1 cell population (10–13). For example, the expression of the CD5 surface glycoprotein divides B-1 cells into B-1a (CD5+) and B-1b (CD5-) (14, 15).

Defining the phenotype of B-1 cells in humans has been a topic of debate (16). Human peripheral blood CD5+ B cells share with murine B-1a cells the ability to generate polyspecific autoreactive antibodies, such as rheumatoid factor and anti-ssDNA (17–20). However, the equivalence of human CD5+ B cells to B-1a cells in mice is not clear because CD5 is present on different subsets of human B cells, including activated, pre-naive, and transitional B cells (21–25). In 2011, CD20+CD27+CD43+CD70− B cells were proposed as human B-1 cells because they exhibit functional characteristics similar to those of mouse B-1 cells, such as spontaneous IgM secretion, efficient T cell stimulation, and tonic intracellular signaling (26). Subsequent studies cast doubt on the quantification of these cells, suggesting that they may include other subsets, such as precursors of plasmablasts with a CD20+CD38^hi^ phenotype (27–31). Consequently, the phenotype of human B-1 cells is more accurately represented by CD19+CD20+CD38^low/int^CD27+CD43+ among lymphocytes that are negative for T cell markers (32, 33). In 2022, Suo et al. conducted a comprehensive characterization of human prenatal B-1 cells using single-cell transcriptomic analysis and B cell receptor (BCR) information, in addition to validating spontaneous antibody secretion functionally. The authors demonstrated that prenatal B-1 cells with the above-mentioned phenotypic markers share distinctive characteristics with mouse B-1 cells and emerge in the early stages of development (34). The implementation of this B-1 cell phenotype in current studies is gaining acceptance and is being used in translational studies of specific disease states (35–44).

The best-known function of B-1 cells is the secretion of natural antibodies (NAbs) (45). NAbs are constitutively produced by B-1 cells even in the absence of deliberate antigenic stimulation and in germ-free animals (46–48). Following innate stimulation by toll-like receptor (TLR) agonists or microbial pathogens, B-1 cells secrete large amounts of antibodies, mainly IgM, which are critical for fighting infections (49). These features position B-1 cells as key players in the first line of defense against pathogens such as viruses, bacteria, and parasites. B-1 cell-derived antibodies are often autoreactive (48, 50, 51) and bind to antigens such as phosphorylcholine (PC) (52, 53), phosphatidylcholine (PtC) (54), and oxidized LDL (OxLDL) (55). Due to this, NAbs serve not only as a first line of defense against invading pathogens but also perform homeostatic housekeeping functions, including the removal of apoptotic cells and the reduction of inflammatory responses (56, 57).

Apart from natural and induced antibody secretion, B-1 cells are known to play crucial roles in phagocytosis, antigen presentation (58), T cell activation (59), and immune regulation through interleukin-10 (IL-10) secretion (60). Some of the functions of B-1 cells have made them a focus of research in the field of autoimmunity. Mounting evidence suggests that B-1 cells are involved in the development of autoimmune diseases such as multiple sclerosis (MS) (61) and systemic lupus erythematosus (SLE) (62).

In addition to their involvement in infectious and autoimmune diseases, a new wave of research has described an emerging role of B-1 cells in influencing tumor growth and anti-tumor immune responses. In this review, we focus on the dual role of B-1 cells in cancer progression. The recent findings will help us understand the full potential of B-1 cells in tumor immunology and will contribute to exploring potential therapeutic strategies involving the B-1 cell population.

## 3 B-1 cells infiltrate tumors

B-1 cells in mice are primarily located in the peritoneal and pleural cavities, but they can also be found in the spleen and bone marrow, with minimal presence in lymph nodes and blood. Recently, Suchanek et al. (63) demonstrated that B-1 cells are also present in major non-lymphoid organs under normal physiological conditions, including the kidney, urinary bladder, liver, and lung. Furthermore, various types of stimulation, such as TLR ligands, induce a massive egress of B-1 cells from the peritoneal cavity and their subsequent migration to other anatomical sites. For example, injection of lipopolysaccharides (LPS) (64) or bacteria (65) induces the rapid migration of B-1 cells from the peritoneal cavity and their accumulation in the omentum, mesenteric lymphoid tissues, and spleen. The activation of B-1 cells resulted in MyD88-dependent changes in the surface expression of integrins, such as CD9, suggesting that the effect was mediated by innate signals (65). Similarly, following influenza or nematode infection, B-1 cells from the pleural cavity were observed to accumulate in the draining mediastinal lymph nodes (66) and in local mediastinal fat-associated lymphoid tissues (67), respectively. Together, these results show that B-1 cells are a dynamic population that, under certain conditions, migrate from their regular reservoir site to other tissues to fulfill their function.

Tumor-infiltrating CD4+ and CD8+ T cells usually express the B cell-recruiting C-X-C motif chemokine ligand 13 (CXCL13), possibly to request B cells help (68–70). Compared to B-2 cells, B-1 cells express higher levels of the CXCL13 receptor, CXCR5, and are more sensitive to CXCL13. B-1 cells exhibit a strong chemotactic response to CXCL13, both *in vitro* and *in vivo* (71). For example, B-1 cells migrate to the omentum and peritoneal cavity in a manner dependent on CXCL13, following the secretion of this chemokine by cells in the omentum and peritoneal macrophages. CXCL13-deficient mice exhibit decreased natural anti-PC antibodies production and an impaired response to bacterial antigens in the peritoneum (71). However, there is currently no evidence as to whether B-1 cells could be recruited to the tumor in a CXCL13-dependent manner. On the other hand, Vivanco at al (72). suggested that other chemokines may be relevant for recruiting B-1 cells into the TME. According to these authors, approximately 10-14% of peritoneal B-1 cells express CCR5 on their surface and migrate in a CCR5-dependent manner in response to contact-independent stimuli from B16F10 melanoma cells.

In recent years, an increasing number of studies have shown that B-1 cells are indeed infiltrating tumors. Kobayashi et al. reported the presence of tumor-infiltrating CD19+CD5+CD43+ B-1a cells in B16F10 melanoma (73). Additionally, Shibad et al. (74) demonstrated that a subset of B-1 cells expressing programmed death ligand 2 (PD-L2) accounted for up to 18% of the total number of B cells in melanoma tumors. A recent report demonstrated that B-1 cells are not only present in B16F10 primary tumors but also infiltrate cutaneous metastasis (75). In addition to melanoma, B-1 cells also infiltrate E0771 mammary tumors in mice (76). While these studies have demonstrated the presence of B-1 cells infiltrating the tumors, the specific anatomical localization and the factors influencing B-1 cell function within the TME remain unknown. In the following two sections, we will discuss the potential mechanisms by which B-1 cells may promote or inhibit tumor progression (**Table 1**).

**Table 1:** Dual role of B-1 cells in cancer.

Function	Phenotype	Mechanism	Effect	Setting	References
Pro-tumor	Mouse B-1a CD19+CD5+CD43+	Secrete IL-10 which in turn, induce a decrease in the proportion of tumor-infiltrating CD8+T cells secreting IFN-γ and TNF-α	Increase tumor growth and reduce survival	B16F10 melanoma	(73)
	Mouse B-1 CD19+CD11b+	B-1 cell direct contact with melanoma cells increases ERK signaling and metastatic potential	Increased lung nodules	B16 melanoma	(77, 78)
	Mouse B-1b CD19^hi^PD-1+CD11b+ CD5-CD21/35^lo^	PD-1+ B-1 cells suppress CD4+ T cell-dependent specific antibody responses against tumor-associated carbohydrate antigens	Reduce survival	TA3-Ha model of peritoneal carcinomatosis	(79)
Anti-tumor	Mouse peritoneal B-1 CD19+CD23−	Unknow	Inhibit tumor growth and confer concomitant immunity	Ehrlich’s carcinoma	(80)
	Mouse peritoneal B-1a CD5+	Secrete tumor-reactive IgM that induces tumor cell killing	Reduce tumor growth, ascites development, and mortality	TA3-H murine mammary carcinoma	(81)
	Mouse B-1a PD-L2+ (termed “L2pB1”)	L2pB1-derived IgMs bind and induce tumor cell apoptosis	Inhibit tumor growth	B16F10 melanoma and MC38 colon cancer tumor	(74)
	Phenotype not defined	Natural antibodies recognize and eliminate neoantigen-expressing precancerous cells by recruiting different immune cells to the tumor site for elimination	Reduce tumor burden and activate anti-tumor immunity	B16F10 melanoma, Lewis lung carcinoma, mouse mammary tumor virus (MMTV)-induced breast tumor, urethane-induced spontaneous lung tumors	(82–84)
	Human B-1 cells from peripheral blood mononuclear cells (PBMC) CD19+CD20+CD38^low/int^CD27+CD43+	Secrete anti-Neu5GcGM3 IgM that binds to and eliminates malignant cells expressing Neu5GcGM3 tumor antigen through complement-dependent and independent mechanisms.	Kill tumor cells *in vitro*	Present in healthy subjects	(85–87)
	Mouse B-1a cells 4-1BBL+ (termed “4BL”)	Induce antigen-specific antitumor GrB+CD8+T cells	Retard tumor growth	B16F10 melanoma	(88)

## 4 The pro-tumor activity of B-1 cells

**Figure 1:**
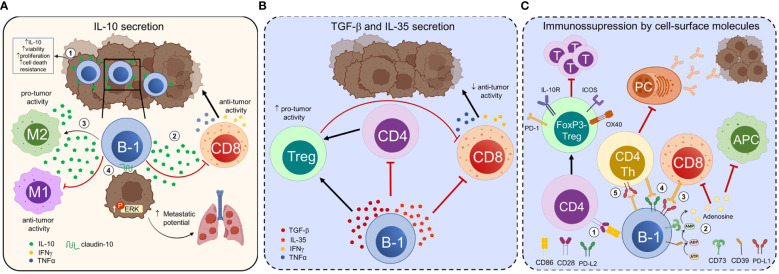
B-1 cells exert immunosuppressive and tumor-promoting functions. B-1 cells regulate anti-tumor immune responses and modulate tumor cell properties through contact-dependent and -independent mechanisms. **(A)** The interaction of tumor cells with B-1 cells increases their ability to secrete IL-10 and enhances the viability, proliferation, and resistance to cell death of B-1 cells (1). Tumor-infiltrating B-1 cells secrete IL-10, which suppresses the production of IFN-γ and TNF-α by CD8+ T cells, thereby promoting melanoma growth (2). IL-10 secreted by B-1 cells polarizes tumor-infiltrating macrophages towards an alternatively activated pro-tumoral M2-like phenotype and induces their secretion of IL-10, favoring an immunosuppressive microenvironment and tumor progression (3). B-1 cells directly influence the properties of tumor cells. In the presence of endogenous IL-10, B-1 cells express claudin-10. The expression of this integral protein involved in cell-to-cell contact in B-1 cells leads to increased ERK signaling in tumor cells, promoting metastasis (4). **(B)** B-1 cells secrete immunosuppressive cytokines such as TGF-β and IL-35 that promote the development of regulatory T cells (Tregs) and suppress or misdirect effector CD4+ and CD8+ T cell responses. C) CD86 mediates cell-to-cell contact between B-1a cells and naive CD4+CD25- T cells, inducing their differentiation into FoxP3- Tregs, named “Treg-of-B-1a”. Treg-of-B-1a cells upregulate OX40, PD-1, ICOS, and IL-10R and exhibit suppressive functions, such as suppressing effector T cell proliferation (1). CD39 and CD73 ectonucleotidases transform ATP into adenosine in the extracellular environment. Extracellular adenosine suppresses the function of antitumor immune cells and promotes the activity of regulatory immune cells, thus creating an immunosuppressive tumor microenvironment. CD73+ B-1 cells have an immunosuppressive effect on autoimmunity, although their relevance in cancer has not been proven (2). PD-L1 expression on B-1 cells may inhibit the anti-tumor function of effector cells, such as CD8+ cytotoxic T cells (3). CD5+ B-1a cells co-expressing PD-L1 and PD-L2 suppress CD4+ and CD8+ allogeneic T-cell responses (4). PD-1+ B-1 cells suppress CD4+ T cell-dependent specific antibody responses against tumor-associated carbohydrate antigens, promoting tumor progression (5). The mechanisms represented in **(B)** and in (2), (3), and (4) from **(C)** (dotted lines, blue background) are extrapolated from other homeostatic or pathological states and need to be confirmed in cancer. PC, plasma cells; T, T cells.

During tumor progression, malignant cells secrete and express several molecules that contribute to modifying the immune microenvironment within the tumor. These changes in the immune populations, in turn, promote tumor growth, angiogenesis, metastasis, and suppression of active immune responses (89, 90). Much of this effect is attributed to the recruitment and expansion of pro-tumor macrophages, myeloid suppressor cells, regulatory T cells (Tregs), and Bregs. Recently, B-1 cells have been shown to exhibit immunosuppressive functions that may undermine anti-tumor immunity or promote tumor progression (**Figure 1**).

### 4.1 IL-10 secretion is the best-known mechanism of B-1 cell-mediated immunosuppression

IL-10 is a regulatory cytokine that plays a crucial role in the modulation of immune responses. Since the early 1990s, it has been shown that mouse peritoneal CD5+ B-1a cells spontaneously secrete IL-10, and the production of IL-10 increases upon innate stimulation with LPS (91). These results raised the possibility that B-1 cells may have immunoregulatory activity. Subsequent studies have shown that other TLR agonists and certain viruses, such as influenza and baculovirus, also efficiently induce IL-10 production by neonatal CD5+ B-1a cells. For example, upon TLR9 stimulation, neonatal B-1a cells produce high levels of IL-10, which prevents optimal IL-12 secretion by conventional dendritic cells (DCs), thereby suppressing Th1 priming (92). Peritoneal cavity CD5+ B-1a cells also secrete high amounts of IL-10 after combined activation with LPS and anti-CD40 (93). In a suppression assay, CD40/TLR4-activated peritoneal cavity B cells inhibit the secretion of tumor necrosis factor-alpha (TNF-α) and interferon-gamma (IFN-γ) by CD4+ T cells. Furthermore, other signals, such as type I interferons (IFNs) derived from activated innate cells, promote IL-10 secretion by neonatal CD5+ B-1a cells (94). Interestingly, the ability of B-1 cells to secrete IL-10 increases after being co-cultured with B16F10 melanoma cells or tumor-conditioned medium (95). The interaction with B16F10 cells also enhances the viability, proliferation, and resistance to cell death of B-1 cells. These results demonstrate the ability of tumor cells to alter the function of B-1 cells towards an immunosuppressive phenotype. The mechanisms underlying this functional transition, as well as evidence of similar activity in other mouse or human tumors, have yet to be investigated.

IL-10+ B-1 cells are not only present in the mouse peritoneal cavity, but also found in other tissues, where they exert immunomodulatory functions. For example, B-1 cells are present in the skin of both mice and humans, where they secrete IL-10 (96). In 2019, Kobayashi et al. demonstrated that CD19+CD5+CD43+ B-1a cells infiltrate mouse melanoma and secrete IL-10 upon stimulation (73). Furthermore, the adoptive transfer of B-1a cells from wild-type mice, rather than IL-10- deficient mice, resulted in a significant increase in tumor growth. The authors have identified that tumor-infiltrating B-1a cells promote melanoma growth by suppressing the production of IFN-γ and TNF-α by CD8+ T cells through an IL-10-dependent mechanism. Further studies are necessary to investigate the presence of IL-10+ B-1 cells infiltrating other tumors.

In addition to their immunosuppressive action on T cells, B-1 cells also regulate the function of other immune cell populations. For example, the IL-10 secreted by B-1 cells reduces the phagocytic capacity of macrophages *in vitro* by decreasing their production of nitric oxide and hydrogen peroxide (60). In co-culture studies involving B-1a cells and macrophages, the production of pro-inflammatory mediators such as IL-6 and TNF-α was lower, while the production of IL-10 was higher compared to macrophage monocultures. This effect was described as IL-10 dependent, as the co-culture of IL-10-deficient B-1a cells and wild-type macrophages did not reduce the level of the pro-inflammatory cytokines (97).

In a separate study, Wong et al. (98) demonstrated that B-1 cells, when co-cultured with peritoneal macrophages, polarize them to a phenotype characterized by reduced expression of pro-inflammatory genes (such as *Tnfa, Ccl3*, and *Il1b*) in response to LPS stimulation, and an increase in the expression of the anti-inflammatory gene *Il10*. In the B16F10 melanoma tumor model, B-1 cells have been shown to polarize the macrophages present in tumors towards an alternatively activated pro-tumoral M2-like phenotype (98). The tumor-associated macrophages (TAM) exhibit reduced expression of the M1 genes *Tnfa, Ccl3*, and *Il1b*, but elevated levels of the M2 markers *Ym1, Fizz1, Il1ra, Mgl1*, and *Mgl2* following the respective stimulation for M1 or M2 phenotype polarization. The authors also demonstrated the direct involvement of B-1-cell-derived IL-10 in promoting IL-10 expression by macrophages and their subsequent M2 polarization (98). M2 macrophages produce immunosuppressive cytokines that exert pro-tumoral functions within the TME (99, 100), suggesting that B-1 cells may indirectly promote tumor progression through this mechanism.

B-1 cells not only modulate other immune cells but also, under certain conditions, have the ability to directly influence the properties of tumor cells. When B16F10 melanoma cells were co-cultured with B-1 lymphocytes, an increase in the activation of the phospho-extracellular signal-regulated kinase (ERK) signaling pathway in the tumor cells was observed. The increased phosphorylation of ERK induced by B-1 cells regulates the expression of genes associated with metastasis, such as *matrix metalloproteinase-9* (*MMP-9*) and the chemokine receptor *CXCR4*, in B16F10 cells (101). In this way, ERK signaling subsequently led to a higher number of lung metastases after the tumor cells were injected into C57BL/6 mice (77, 101). Additionally, the depletion of B-1 cells in the peritoneal cavity significantly reduced the number of lung metastases (77). The pro-metastatic effect of B-1 cells on B16F10 melanoma cells depended on cell-to-cell contact. According to Perez et al. (78), in the presence of endogenous IL-10, B-1 cells express claudin-10, an integral protein involved in cell-to-cell contact. This interaction leads to increased ERK signaling in B16F10 cells, promoting metastasis.

In humans, a small proportion of B-1 cells express the CD11b marker. In contrast to CD11b- B-1 cells, this particular subset secretes IL-10, suppresses CD3-mediated T-cell activation, and is significantly elevated in patients with lupus (102, 103). However, it is unknown whether this population is involved in suppressing anti-tumor immunity.

### 4.2 Secretion of immunosuppressive cytokines other than IL-10

#### 4.2.1 Transforming growth factor-β

Besides IL-10, various cytokines have been implicated in suppressing anti-tumor immunity, including transforming growth factor-β (TGF-β) and interleukin-35 (IL-35). TGF-β is a highly prevalent soluble factor in the TME. This cytokine can be produced by tumor cells and immune cells, such as Tregs and B cells with regulatory function (104). TGF-β secreted by tumor-evoked Bregs (tBregs) induces the conversion of naive CD4+ T cells into FoxP3+ Tregs in a 4T1 mammary tumor model (105). Similarly, EMT-6 mammary tumor-educated B cells suppress the activation of CD4+ and CD8+ T cells through a TGF-β-dependent mechanism (106). In a murine model of hepatocellular carcinoma, IgA+ B cells expressing PD-L1, IL-10, and TGF-β suppressed the proliferation and activation of CD8+ T cells (107). Xiao et al. (108) recently demonstrated the capacity of B-1 cells to produce TGF-β following *Schistosoma japonicum* infection. The authors reported that following *in vitro* stimulation with soluble egg antigens (SEA), splenic B-1a cells, but not splenic CD5- B cells, exhibited significantly increased expression of TGF-β. Furthermore, both B-1a cells and CD5- B cells in the peritoneal cavity showed a significant increase in the expression of TGF-β after stimulation with SEA. While these results demonstrate the ability of B-1 cells to produce TGF-β under specific conditions, it is still necessary to clarify whether this mechanism occurs in cancer.

#### 4.2.2 IL-35

IL-35 is a member of the IL-12 family and plays significant roles in the regulation of tumor immunity (109). The immunosuppressive function of IL-35 was first identified in the context of autoimmune and inflammatory conditions (110, 111) such as autoimmune uveitis and experimental autoimmune encephalomyelitis (112, 113). Beyond the scope of autoimmune disease, a specific subset of B cells with a CD1d^hi^CD5+ phenotype has been found to produce IL-10 and IL-35 in a mouse model of pancreatic cancer (114). IL-35 transmits signals to CD8+ T cells, thereby augmenting STAT3 activity within these cells. The activation of STAT3 suppresses the expression of chemokine receptors CXCR3 and CCR5, as well as the expression of the effector cytokine IFNγ in CD8+ T cells. This leads to a reduced capacity of CD8+ T cells to infiltrate tumors and generate effective responses. To date, IL-35–producing Bregs have been primarily thought to originate from terminally differentiated plasma cells (112, 113). However, Choi et al. (115) demonstrated that human umbilical cord blood contains a small proportion of IL-35–producing Bregs derived from B-1 cells, which increase by more than two-fold following BCR activation. Further research is necessary to explore the immunosuppressive effect of this particular B-1 subset and to demonstrate their participation in cancer progression.

### 4.3 Immunosuppression resulting from the expression of molecules on the surface of B-1 cells

The production of cytokines is just one of the many mechanisms by which B-1 cells suppress immune responses. The inhibitory functions of B-1 cells may also be attributed to mechanisms that involve the expression of specific molecules on their surface and/or direct interaction with target cells. For example, although mouse B-1 cells do not exhibit a preference for inducing FoxP3+ Tregs (59), they do induce FoxP3- Tregs via an IL-10 independent immunosuppressive mechanism, which warrants further investigation. B-1a cells have the ability to transform naive CD4+CD25− T cells into a distinct subset of T cells referred to as “Treg-of-B-1a,” which do not express FoxP3 but exhibit suppressive function. Instead, Treg-of-B-1a cells upregulate the Tregs markers OX40, programmed cell death protein 1 (PD-1), inducible costimulator (ICOS), and IL-10R (116). This subset of Tregs exerts suppression by secreting soluble factors that have yet to be defined. While the secretion of IL-10 by B-1 cells is not involved in the induction of Treg-of-B-1a cells, it was found that cell-to-cell contact and CD86-mediated costimulation were crucial for their induction.

#### 4.3.1 CD73

Adenosine, a nucleoside derived from adenosine triphosphate (ATP), accumulates within the TME and significantly contributes to tumor evasion. ATP is released upon cell lysis and is subsequently broken down to adenosine by the ectonucleotidases CD39 and CD73, which are present on the surface of tumor cells and immune cells within the TME. Adenosine reduces the activation, proliferation, and anti-tumor activity of CD8+ T cells by acting on the A2A and A2B receptors present in T cells and antigen- presenting cells (APC), respectively (117). B cells in mice typically express CD39, while CD73 expression is not commonly observed. In 2014, Kaku et al. (10) reported that approximately 30–50% of the total B-1 cells express high levels of CD73. In contrast, only a small fraction of B-2 cells exhibit markedly low levels of CD73 expression. CD73-positive B-1 cells have the capacity to generate adenosine in the presence of substrate, and they can alleviate the severity of colitis, indicating the immunosuppressive role of this particular cellular subset. Further studies are necessary to determine whether B-1 cells expressing CD73 suppress tumor immunity and promote tumor progression.

#### 4.3.2 PC1

In addition to CD39, ATP undergoes hydrolysis by other enzymes present on the cell surface, including plasma cell alloantigen 1 (PC1). In mice, B-1a cells exhibit distinctive innate immune and immunoregulatory functions, which are influenced by their surface expression of PC1 (12). Upon adoptive transfer, PC1^lo^ cells secrete higher levels of circulating natural IgM, whereas PC1^hi^ cells produce more IL-10 compared to their PC1^lo^ counterparts. As a result, PC1^hi^ cells exert a negative regulatory effect on the differentiation of Th1 cells. It is unclear whether tumors contain PC1^hi^ cells or if the IL-10 they produce plays a role in suppressing the immune response to tumors. In the future, it would be interesting to explore whether B-1 cells infiltrating the tumor express CD73 or PC1, thereby contributing to the tumor suppressive microenvironment.

#### 4.3.3 PD-1/PD-L1, PD-L2

PD-1 is an inhibitory receptor expressed in activated monocytes, DCs, natural killer T cells, B cells, and T cells. PD-L1 and PD-L2 are the ligands for PD-1. PD-L1 is extensively expressed by many immune and non-immune cells, whereas PD-L2 is restricted to antigen-presenting and regulatory cells (118). Engagement of PD-1 with either of its ligands delivers inhibitory signals that reduce receptor-mediated cell survival, differentiation, and the secretion of pro-inflammatory cytokines. The PD-1 signaling pathway has been shown to inhibit immune responses and promote self-tolerance (119). PD-L1 and PD-L2 can also transmit inhibitory signals in the opposite direction when interacting with PD-1 or CD80. PD-1 and PD-L1 exhibit high expression levels on Tregs and Bregs and have been involved in their suppressive activities (120, 121).

The expression of PD-L1 on B cells plays a crucial role in regulating both T and B cell responses (122). Bregs with PD-L1 expression exert a significant immunosuppressive effect in various tumors (reviewed in (123) (124)). In Balb/c mice, naive B cells in the peritoneal cavity exhibited significantly higher levels of PD-L1 compared to B cells in other organs. Among the B cells, the CD11b+CD5+ B-1a subset exhibits the highest expression, with over 90% of the cells expressing PD-L1 (125). However, the suppressive function of PD-L1+ B-1a cells on tumor immunity requires further study.

PD-L2 is expressed in various human cancers. Similar to PD-L1, it significantly suppresses antitumor immune responses, including those of tumor antigen-specific CD8+ T cells (126, 127). Certain subpopulations of memory B cells can be characterized based on their CD80 and PD-L2 expression (128). Memory B cells expressing both CD80 and PD-L2 markers (CD80+PD-L2+) undergo rapid differentiation into antibody-forming cells but do not give rise to germinal centers, unlike CD80-PD-L2- memory B cells. The authors of the study hypothesize that the expression of CD80 and PD-L2 may modulate T cell responses and, consequently, the functions of memory B cells. Approximately 50% of murine B-1a cells express PD-L2 (13), suggesting that this specific subset of cells may also possess a distinct regulatory function. McKay et al. (129) demonstrated that the expression of PD-L2 regulates the production of NAbs against PC and PtC. Mice lacking PD-L2 exhibited elevated levels of anti-PC IgM and a notable increase in PC-reactive plasmablasts with a B-1a phenotype. The increase in PC-specific humoral response in these mice was correlated with enhanced protection against a constitutively PC-expressing mutant of non-typeable Haemophilus influenzae (NTHi). The authors subsequently suggest that the expression of PD-L2 in B-1 cells hinders their capacity to differentiate into antibody-secreting cells by regulating T cell secretion of IL-5, a potent stimulator of B-1 cell antibody production.

B-1 cells that express both PD-L1 and PD-L2 also have the capacity to inhibit alloimmune T- cell responses. The injection of B cells from the peritoneal cavity of Balb/c, which contained B-1a cells co-expressing PD-L1 and PD-L2, into C57BL/6 mice, resulted in a notable decrease in the levels of anti-Balb/c antibodies and in T cell responses to Balb/c-allostimulation. The study demonstrated that B-1 cells expressing PD-L1 and PD-L2 can suppress T cells in response to cognate allostimulation (125). However, in tumor models, PD-L2-positive B-1 cells seem to play an anti-tumoral role rather than an immunosuppressive function (74). This effect will be further discussed in the following section.

PD-1 has been implicated in the regulation of the antibody response to T cell-independent type 2 antigens (TI-2 Ag). PD-1 is expressed by antigen-specific B cells, such as B-1b cells, shortly after immunization with TI-2 Ag (130, 131). In 2011, Haas used an anti-PD-1 blocking monoclonal antibody to interfere with PD-1/PD-L1 interactions while immunizing with TI-2 Ag (132). Through this strategy, the author has demonstrated that the interaction between PD-1 and PD-L1 suppresses the expansion of antigen-specific B-1b cells, isotype switching, and antibody production in response to TI-2 Ags. Later, Haro et al. (79) demonstrated that the intrinsic expression of PD-1 in B-1b cells hinders the development of humoral immune responses that provide protection against tumors carrying the Thomsen-nouvelle/CD175 antigen (Tn), a tumor-associated carbohydrate antigen (TACA) (133). The collective findings suggest that PD-1 expression plays a significant role in regulating B-1 cell antibody responses, including tumor-specific humoral responses. In humans, the levels of naturally-occurring antibodies with reactivities attributed to B-1 cells decrease in cancer patients compared to healthy donors (85, 86, 134). While a mechanism to account for this observation has not yet been elucidated, one possibility is that B-1 cells exhibiting these reactivities may be subject to immunosuppression in cancer patients, potentially involving the PD-1/PD-L1 axis.

**Figure 2:**
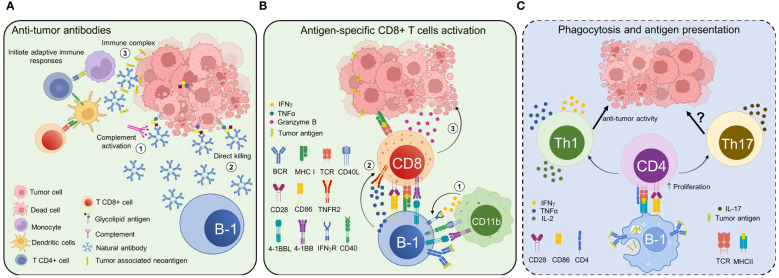
Potential anti-tumor roles of B-1 cells and natural antibodies. B-1 cells perform their diverse anti-tumor roles through both antibody-dependent and antibody-independent mechanisms. **(A)** Natural antibodies secreted by B-1 cells recognize tumor-associated antigens and induce tumor cell death through various mechanisms, such as complement-dependent (1) and -independent cytotoxicity (2). B-1 cell-derived IgM forms immune complexes with neoantigens derived from tumor cells and activates adaptive immunity to remove transformed cells at a precancerous stage (3). **(B)** B-1a cells could give rise to 4BL cells, which in turn induce cytotoxic CD8+ T cells. Under certain conditions, CD11b+ myeloid cells and/or macrophages can convert B-1a cells into 4BL cells expressing 4-1BBL, TNFα, IFNγR, and major histocompatibility complex class I molecules. Then, 4-1BBL and IFNγR signaling further increase TNFα and CD86 expression on 4BL cells (1), which activate CD8+ T cells to express 4-1BB and TNFR2 (2). TNFα signaling through TNFR2 and the co-stimulation with CD86 induce the expression of Granzyme B in CD8+T cells (3), which ultimately inhibits tumor growth. **(C)** B-1 cells can phagocytose antigens and function as antigen-presenting cells, although the relevance of this mechanism has not been proven in cancer (represented in a panel with dotted lines and blue background). B-1 cells can present antigens acquired through their surface receptors or by phagocytosis. Further, they can activate adaptive immune responses by polarizing CD4+ T cells to a pro-inflammatory T helper 1 (Th1) or T helper 17 (Th17) phenotype. The increased CD86 expression on B-1 cells contributes to their strong antigen presentation and efficient T-cell stimulation.

In the following section, we will examine the mechanisms employed by B-1 cells to fight tumors (**Figure 2**). These mechanisms may coexist with or operate independently of the immunosuppressive mechanisms previously discussed. The prevalence of one type of B-1 cell response over the other could potentially impact the outcome of cancer. Nevertheless, extensive research is still required to elucidate the role of B-1 cells in tumor immunity.

## 5 The anti-tumor function of B-1 cells

### 5.1 The secretion of tumor-reactive NAbs

B-1 cells have the ability to exert anti-tumor activity by secreting NAbs that specifically target tumor-associated antigens (TAA) in both mice and humans. Reports documenting NAbs’ role in host-mediated natural resistance against neoplasia began to emerge in the 1980s. The initial investigations used Xid mice as an experimental model to examine the *in vivo* role of NAbs. Xid animals exhibit a mutation in the gene encoding the protein Bruton tyrosine kinase (Btk), leading to a nearly complete absence of CD5+ B-1 cells. This is accompanied by a significant decrease in the levels of circulating IgM and IgG3 (135). In 1989, Chow and Bennet (136) conducted a study that showed an increased tumor burden in Xid mice compared to their normal counterparts. Furthermore, serum derived from normal mice exhibited greater binding and complement dependent cytotoxicity (CDC) against tumor cells compared to serum from Xid mice. The above-mentioned findings suggest a potential anti-tumor function for NAbs. However, it is noteworthy that in Xid mice, not only B-1 cells but also other mature B-cell populations, including IgM^lo^IgD^hi^ splenic B cells, are affected (137).

To more effectively evaluate the *in vivo* anti-tumor activity of NAbs, Chow et al. conducted a study in which they examined the effects of intravenous administration of serum polyclonal NAbs from normal mice to Xid mice (138). The tumorigenicity of T-cell lymphoma RI-28 significantly decreased when Xid mice were reconstituted with whole syngeneic serum NAbs, serum NAbs precipitated with ammonium sulfate or a combination of purified IgG and IgM NAbs from normal mice.

In line with the results of prior research, Azevedo et al. (80) presented evidence demonstrating the involvement of B-1 cells in concurrent immunity within the Ehrlich tumor model. Balb/c mice rejected a secondary tumor implant, while Xid mice developed secondary tumors. Adoptive transfer of purified B-1 cells from naive Balb/c mice reinstated tumor-associated immunity. The authors also provided evidence to support the notion that B-1 cells originating from mice with Ehrlich tumors can confer protection against the development of the same type of cancer in recipient mice.

Recently, additional transgenic mouse models have been employed to further clarify the role of NAbs in identifying and eliminating neoantigen-expressing tumor cells, including those found in the precancerous stage. For example, the sera obtained from naive AID-/- mice with a hyper-IgM repertoire exhibited strong binding to MB16F10 melanoma cells, while no binding was observed with sera obtained from Rag2-/- mice, who lack antibodies (82).

Díaz-Zaragoza et al. (139) performed an investigation into the tumor-binding properties of natural IgMs obtained from three different mouse strains that exhibit varying susceptibility to spontaneous breast cancer development. The results showed significant differences in the recognition of tumor antigens among the three strains, affirming the presence of strain-specific repertoires of natural IgM antibodies. Hence, both the quantity and diversity of natural IgM play a role in controlling tumor progression. To support this notion, a series of studies were conducted on transgenic animals lacking a complete repertoire of NAbs. IghelMD4 mice, characterized by over 90% of IgM-secreting B cells specific to chicken egg lysozyme, exhibited a greater tumor burden compared to wild-type (WT) mice in various tumor models (82–84).

To demonstrate the primary role of circulating NAbs in contrast to those expressed in B cell membranes in the process of binding and eliminating tumor cells, Rawat et al. (82) established a mouse model with a deficiency in circulating NAbs. The authors developed a mouse model lacking circulating IgG and IgM antibodies while preserving membrane-bound IgM by crossbreeding hyper-IgM Aicda -/- mice with sIgM -/- mice. Following the inoculation of both the transgenic mice and their wild-type counterparts with B16F10 melanoma cells, the transgenic mice exhibited a significantly higher number of lung nodules compared to the wild-type mice. This observation confirms the significant role of circulating NAbs in anti-tumor immunity.

The mechanisms by which NAbs eliminate tumor cells have been extensively reviewed (140). In summary, NAbs have the ability to eliminate tumor cells through diverse mechanisms, such as ADCC, CDC, and complement-independent cytotoxicity (CIC). Beyond their conventional effector functions, natural IgMs are essential for the recognition of tumor neoantigens and the initiation of an adaptive immune response against tumor cells. Attif et al. (83) demonstrated that natural IgM antibodies have the capability to form immune complexes with neoantigens derived from tumor cells. This interaction subsequently enables monocytes to present neoantigens as immunogens to CD4+ T helper cells. This, in turn, enables the licensing of DC to cross-present and elicit a cytotoxic T cell response against tumor cells expressing neoantigens. Pathogen-associated molecular patterns (PAMPs) are typically necessary to license APC for the presentation of antigens as immunogens, but they are not frequently present during the initial phases of carcinogenesis. This fact highlights the importance of NAbs in eradicating early-stage tumors in the absence of PAMPs.

Although NAbs can provide a first line of defense against cancer cells in the absence of PAMPs, the presence of these danger signals, which non-specifically activate B-1 cells, helps overcome immune suppression and enhances anti-tumor responses (141). For example, Haro et al. (81) demonstrated that the co-administration of monophosphoryl lipid A (MPL) and trehalose-6,6’-dicorynomycolate (TDCM) effectively suppresses tumor growth and ascites formation in murine models of peritoneal carcinomatosis and lymphomatosis. The observed anti-tumor effect required B-1 cells to secrete high levels of natural IgM specific to TACAs, along with the activation of the classical complement pathway. Mucin-associated antigens, such as Thomsen-Friedenreic (TF), Tn, sialyl Tn, and ganglioside GD2, are TACAs that have the potential to be recognized by IgM antibodies produced by B-1 cells following MPL/TDCM treatment.

The NeuGcGM3 ganglioside is considered an intriguing example of TAA. Humans lack the ability to synthesize NeuGc gangliosides due to a genetic mutation in the cytidine monophosphate-N-acetyl-neuraminic acid hydroxylase (*cmah*) gene (142). This gene encodes an enzyme that converts N-acetyl to N-glycolyl sialic acid. Despite the inactivation of *cmah*, several studies have shown the overexpression of NeuGc conjugates, particularly NeuGc gangliosides such as NeuGcGM3, in various human tumors (143). The screening of immortalized CD5+ hybridomas, derived from peritoneal B cells of a naive Balb/c mouse, revealed that some of the secreted IgMs were able to recognize NeuGcGM3 ganglioside and tumor cells expressing this antigen (134). Moreover, these antibodies induce tumor cell death through both CDC and CIC mechanisms. The findings presented in this study provide support for the idea that tumor-reactive natural IgM antibodies can eliminate tumor cells, thus aiding in tumor immunosurveillance.

Studying NAbs in humans poses significant challenges because individuals are continuously exposed to foreign antigens. However, a body of literature suggests that human NAbs have the potential to eliminate cancer cells during the early stages of the disease (144). Devarapu et al. (145) identified naturally occurring IgM antibodies that can target and eliminate melanoma and neuroblastoma cells in a subset of the healthy Western population. The naturally occurring antibodies were found to recognize gangliosides, such as GD2 and GD3, and to exert their cytolytic activity through a complement-dependent mechanism.

As previously observed in mice, human B-1 cells with the CD19+CD20+CD38^low/int^CD27+CD43+ phenotype also demonstrate the capacity to secrete anti-NeuGcGM3 antibodies (86). The anti-NeuGcGM3 antibodies have the ability to bind to and eliminate tumor cells that express this ganglioside (85, 86, 134). Naturally occurring cytotoxic anti-NeuGcGM3 antibodies have been identified in a significant portion of the healthy human population (85). Nevertheless, there is a notable decrease in both the levels and cytolytic activity of these antibodies as individuals age. This decrease is associated with a reduction in both the quantity and functionality of B-1 cells (26, 87). Taken together, these findings indicate that the age of individuals could potentially influence the immunosurveillance function of naturally occurring antibodies in the human body.

Certain NAbs isolated from cancer patients exhibit the capability to eliminate tumor cells. Some of these NAbs have been evaluated in clinical trials, demonstrating promising results. These monoclonal antibodies (mAbs) are all fully human IgMs and are encoded in the germline. The antibodies specifically target carbohydrate epitopes that are overexpressed on tumor cells, ultimately resulting in the death of the cells. PAM-1 is a monoclonal IgM obtained from a patient with gastric cancer through the application of human hybridoma technology. PAM-1 binds to a tumor-specific N-linked carbohydrate epitope found on a post-transcriptionally modified variant of the cysteine-rich fibroblast growth factor receptor (CFR-1), which is expressed in almost all epithelial cancers. This specific antibody induce apoptosis in adenocarcinoma cells, both *in vitro* and *in vivo*, by efficiently inhibiting the signaling of CFR-1 (146–148). Another example is the mAb PAT-SC1, originally designated as SC-1, which was derived from a patient diagnosed with signet ring cell carcinoma of the stomach (149). The epitope identified by PAT-SC1 is a carbohydrate found on the decay acceleration factor-B (DAF/CD55), a protein overexpressed on the membrane of gastric cancer cells. PAT-SC1 triggers the cross-linking of CD55, resulting in the apoptosis of tumor cells (150, 151). In a ten-year follow-up academic clinical trial, a single administration of PAT-SC1 before gastrectomy was observed to be safe and led to tumor cell apoptosis, ultimately contributing to improved survival outcomes (152). An additional clinically effective NAb is PAT-SM6. This mAb specifically binds to an isoform of the cell surface-associated chaperone, glucose-regulated protein 78 (GRP78), which is primarily expressed in malignant cells. PAT-SM6 induces the lethal accumulation of oxidized lipoproteins, leading to apoptosis of tumor cells (153). A preliminary single-dose clinical trial conducted in patients with in-transit cutaneous melanoma demonstrated the safety and tolerability of PAT-SM6. Histological analysis revealed the localization of PAT-SM6 within the tumor and its ability to induce apoptosis (154). Furthermore, a Phase I trial employing dose escalation was carried out on patients with relapsed and refractory multiple myeloma (RRMM) to assess the safety and effectiveness of PAT-SM6 when used as a standalone treatment. The research results, as reported by Rasche et al. (155), validated the safety of PAT-SM6 and showed a disease stabilization rate of 33%. A subsequent case report showcased partial remission in a patient diagnosed with relapsed and refractory multiple myeloma (RRMM) after receiving treatment with PAT-SM6 in combination with novel agents (156).

Collectively, the aforementioned findings suggest that NAbs have the potential to eliminate tumor cells at various stages, in both preclinical and clinical settings. As previously stated, NAbs primarily target antigens, such as glycolipids, that remain unchanged despite genetic mutations. As a result, their application in clinical settings is anticipated to offer advantages in addressing the challenge of tumor heterogeneity, a factor frequently associated with the emergence of drug resistance. Several approaches to enhance natural IgM levels have been previously discussed by Kaveri et al. (157). In the same line, Haro et al. (81) have proposed an alternative method to enhance the antibody-mediated anti-tumor impact of B-1 cells. The administration of TLR and C-type lectin receptor agonists was demonstrated to induce the production of tumor-reactive IgM by B-1 cells. The immune response described was shown to effectively suppress tumor growth and the formation of ascites.

### 5.2 The process of antigen presentation and T cell activation

In addition to secreting NAbs, B-1 cells also can secrete cytokines and act as APC (158). Zhong et al. (59) demonstrated that peritoneal B-1a cells have the ability to present Ovalbumin (OVA) peptide to OVA-responsive transgenic CD4+ T cells, resulting in the stimulation of their proliferation. Moreover, the presentation of antigens by B-1a cells leads to the stimulation of Th1 cytokine secretion, such as IL-2, IFN-γ, and TNF-α, by CD4+ T cells. In most cases, Th1 cells exert anti-tumor activity. They facilitate tumor rejection by indirectly enhancing the effector functions of other immune cells, such as cytotoxic CD8+ T cells (159). However, there is currently no direct evidence to support the idea that B-1 cells have an anti-tumor effect by inducing Th1 cells.

In the same line, Lee-Chang et al. (88) reported that the aging process results in the transformation of B-1 cells into 4BL cells, which, in turn, induce cytolytic Granzyme B (GrB)- expressing CD8+ T cells. The proposed mechanism suggests that myeloid cells stimulate BCR and CD40 signaling, leading to the upregulation of 4-1BBL, IFN-γR1, membrane TNF-α (mTNF-α), CD86, and MHC-I expression in B-1a cells. The resulting B-1a cells function as APCs that induce antigen-specific GrB+CD8+ T-cell responses. In the MB16F10 melanoma model, aged B-1a cells are converted into APC that uptake endogenous tumor antigens and subsequently suppress tumor growth. The inhibition is achieved through the induction of anti-tumor GrB+CD8+ T cells.

Several studies have shown that B-1 cells have the capacity to promote the differentiation of CD4+ T cells into Th17 cells (59, 160, 161). Nevertheless, the influence of Th17 cells on tumor immunity is a topic of debate due to conflicting evidence indicating both their pro-tumor and anti-tumor roles (159). The question of whether B-1 cells can have an anti-tumor or pro-tumor effect by inducing Th17 cells remains to be resolved.

### 5.3 Phagocytosis

In addition to their lymphoid nature, B-1 cells exhibit several characteristics commonly found in macrophages. Both types of cells express myeloid lineage markers, including F4/80, MHC II, and CD80/CD86 (58, 162, 163). B-1 cells have been shown to possess the ability to differentiate into mononuclear phagocytes (164–166). Additionally, they exhibit a notably elevated phagocytic capacity compared to other B cell subtypes. B-1 phagocytes can engulf large particles (>0.5 µm), including apoptotic debris. The process is facilitated through the use of pseudopodia-like cytoplasmic extensions (58, 167). The phagocytic-like B-1 cells have the ability to engulf particles coated with mannose or PtC, as demonstrated by Vo et al. (168). Moreover, previous research has shown that these cells can phagocytize and eliminate parasites (169) and bacteria, both *in vitro* and *in vivo* (170). In murine models, B-1 phagocytes demonstrate migration towards inflammatory sites within the tissues (171, 172). However, the capacity of B-1 phagocytes to act as professional APCs within tumors and their potential to eliminate malignant cells have not been established.

### 5.4 Anti-tumor role of specific B-1 cells subsets

As mentioned above, B-1 cells can be subdivided based on the presence of surface markers, such as PD-L2, with over 50% of B-1 cells expressing PD-L2. Cells that exhibit this specific characteristic are commonly known as L2pB1 cells, and they are known to secrete natural IgM antibodies, predominantly targeting self-antigens (161). Recently, a correlation was established between L2pB1 cells and the enhancement of the anti-tumor immune response. L2pB1 cells actively accumulate within MB16F10 melanoma tumors and their depletion promotes tumor growth and angiogenesis. Moreover, L2pB1 cells secrete IgM antibodies that can bind to both B16F10 melanoma cells and MC38 colon cancer tumor spheroids, leading to tumor apoptosis (74). The involvement of L2pB1 cells in cancer immunosurveillance may have broader implications beyond the production of tumor-reactive natural IgM alone. In tumor-free naive mice, L2pB1 cells exhibit greater APC potency than other B cells. This observation is attributed to L2pB1’s heightened expression of co-stimulatory molecules, including CD80 and CD86 (161, 173). Actually, L2pB1 cells have been found to effectively enhance T cell proliferation and cytokine production (173), even in the presence of PD-L2 expression. Although it has not been proven in cancer, L2pB1 might be able to present tumor antigens, inducing T cell differentiation and proliferation, and therefore contributing to the elimination of cancer cells. However, this hypothesis requires further investigation.

## 6 Conclusions

The role of B-1 cells in host resistance to infections, inflammation, and immune tolerance remains a topic of significant interest. However, there is limited understanding of the function of B-1 cells in immune surveillance against tumors. In this review, new studies are highlighted, which identify a novel emerging role for B-1 cells in either controlling or promoting tumor progression.

In addition to the secretion of anti-tumor NAbs, current studies also focus on identifying novel molecules expressed by B-1 cells to modulate tumor progression. Similar to other immune cell populations, B-1 cells may play either protective or deleterious roles in tumor immunity, depending on the context in which they reside or become activated. The contribution of B-1 cells to tumor immunity may vary depending on the specific tumor type, anatomical site, and their interaction with other components present within the TME. Further investigation into novel cellular interactions involving B-1 cells and other cells within the TME, as well as the factors that contribute to B-1 cell differentiation and trafficking into the tumor, may provide insights into the unresolved questions regarding the association between B-1 cells and cancer pathogenesis. Moreover, a clear analysis of the role of B-1 cell in tumor progression will contribute to the development of innovative strategies for cancer treatment.

## Author contributions

NR-Z: Conceptualization, Investigation, Writing – original draft, Writing – review & editing. AH: Conceptualization, Writing – original draft, Writing – review & editing.
